# Canonical Correlation Analysis in the Study of Cerebral and Peripheral Haemodynamics Interrelations with Systemic Variables in Neonates Supported on ECMO

**DOI:** 10.1007/978-1-4614-4989-8_4

**Published:** 2012-07-21

**Authors:** Alexander Caicedo, Maria D. Papademetriou, Clare E. Elwell, Aparna Hoskote, Martin J. Elliott, Sabine Van Huffel, Ilias Tachtsidis

**Affiliations:** 1 ESAT/SCD, Department of Electrical Engineering & IBBT Future Health Department, Katholieke Universiteit Leuven, Leuven, Belgium; 2 Department of Medical Physics and Bioengineering, University College London, 18 Dickensons Lane, London, WC1E 6BT UK; 3 Great Ormond Street Hospital for Children, London, UK

**Keywords:** ECMO

## Abstract

Neonates supported on extracorporeal membrane oxygenation (ECMO) are at high risk of brain injury due to haemodynamic instability. In order to monitor cerebral and peripheral (muscle) haemodynamic and oxygenation changes in this population we used a dual-channel near-infrared spectroscopy (NIRS) system. In addition, to assess interrelations between NIRS and systemic variables, collected simultaneously, canonical correlation analysis (CCA) was employed. CCA can quantify the relationship between a set of variables and assess levels of dependency. In four out of five patients, systemic variables were found to be less inter-related with cerebral rather than peripheral NIRS measurements. Moreover, during ECMO flow manipulations, we found that the interrelation between the systemic and the NIRS cerebral/peripheral variables changed. The CCA method presented here can be used to assess differences between NIRS cerebral and NIRS peripheral responses due to systemic variations which may be indicative of physiological differences in the mechanisms that regulate oxygenation and/or haemodynamics of the brain and the muscle.

## Introduction

Extracorporeal membrane oxygenation (ECMO) is a life support system for patients with intractable cardio-respiratory failure. Neonates supported on ECMO often suffer from periods of haemodynamic instability, hypoxia and/or hypercapnia. In addition, the ECMO procedure itself may cause physiological changes due to ligation of the major neck vessels, heparinization and haemodilution, which can cause alterations in cerebral blood flow and potentially disrupt autoregulation [1]. Consequently, ECMO patients have increased risk for brain injury with reported abnormal ­neuroimaging ranging from 28 to 52%, depending on the imaging technique used [2].

Several studies have described changes in the cerebral haemodynamics before, during and after ECMO procedure. Liem et al. [1] reported that mean arterial blood pressure (MABP), arterial oxygen saturation (SaO_2_) and partial pressures of oxygen and CO_2_ measured transcutaneously were some of the variables that better explained changes in cerebral total haemoglobin (HbT) measured by NIRS. Ejike et al. [3] reported that the regional cerebral oxygenation presented a negative correlation with arterial partial pressure of CO_2_ (pCO_2_) and no significant correlation with changes in ECMO flow. Papademetriou et al. [4] used dual-channel NIRS system during ECMO flow changes and reported the presence of low frequency oscillations (<0.1 Hz) in peripheral oxyhaemoglobin (HbO_2_), which are not present in cerebral HbO_2_, demonstrating differences between cerebral and peripheral haemodynamics in this patient group.

Several studies have investigated the relationship between spontaneous changes in MABP and cerebral NIRS signals as assessment of brain autoregulation [5–7]. Brady et al. [6] investigated the correlation between NIRS and MABP in paediatric patients undergoing cardiac surgery with cardiopulmonary bypass for correction of congenital heart defects. They found an association between hypotension during cardiopulmonary bypass and impairment of autoregulation. We have also previously [7] studied the relation between MABP and haemoglobin difference (HbD  = HbO_2_  −  HHb, oxy minus reduced haemoglobin) and tissue oxygenation index (TOI  =  HbO_2_/HbO_2_  + HHb) by means of correlation, coherence and partial coherence analysis, and its use in clinical outcome prediction; although higher values were found in the population with adverse clinical outcome, indicating a stronger relation between MABP and HbD/TOI, no strong evidence was established. However, ECMO is a complex procedure and study of the interrelation of haemodynamic variables, only, with MABP may not be sufficient.

In this study we describe the use of canonical correlation analysis (CCA) to investigate the differences between the interrelations in cerebral and peripheral NIRS measurements with the systemic variables in ECMO patients. In our analysis the systemic variables were defined as the independent dataset, while the cerebral and peripheral NIRS measurements were defined as dependent variables.

## Methods

CCA is a statistical method that analyzes the interrelation between variables in ­multidimensional datasets. CCA can be seen as an extension to normal correlation analysis, in which the proximity between two multidimensional datasets, instead of vectors, is analyzed by means of canonical angles [8]. CCA determines how strongly the variables in both datasets are related. It is also possible to determine which and how many of the independent variables explain most of the variation in the dependent dataset.

**Fig. 4.1:**
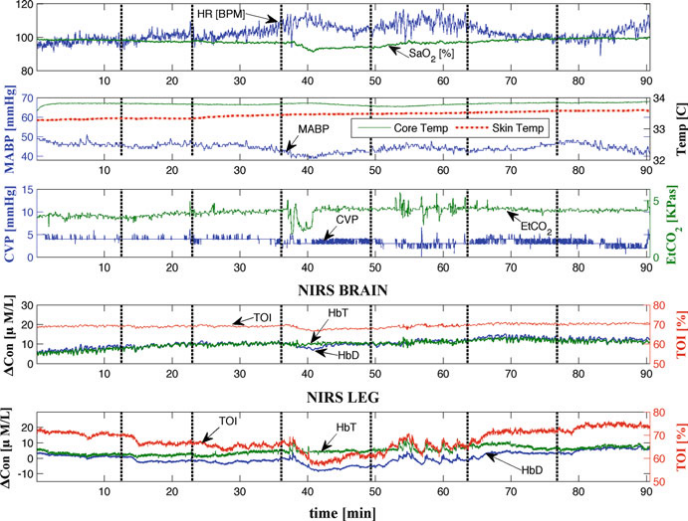
Systemic and haemodynamics measurements recorded from patient 3. The *dashed ­vertical lines* represent the changes in ECMO flow

Measurements from five subjects (ranging from 1 to 1,825 days) on veno-arterial (VA) ECMO procedure were used in this study. A dual-channel near-infrared system (NIRO 200, Hamamatsu Photonics KK) was used to measure the changes in HbO_2_, HHb and TOI using spatially resolved spectroscopy. From these signals HbD and total haemoglobin changes (HbT  =  HbO_2_ +  HHb) were calculated and used, together, with TOI for further analysis. NIRS data were collected at a frequency of 6 Hz. Channel 1 was placed on the forehead in order to assess cerebral NIRS changes, while channel 2 was placed on the calf to assess peripheral NIRS changes. A full set of systemic data including MABP, central venous pressure (CVP), end-tidal carbon dioxide pressure (EtCO_2_), heart rate (HR), respiration rate (RR), core and skin temperatures and SaO_2_ were continuously measured in real time at the bedside (IntelliVue MP70, Philips Medical). All signals were down-sampled to 1 Hz and artefacts were removed manually by means of interpolation. Figure 4.1 shows an example of the systemic and NIRS measurements from one neonate. Measurements were done during stepwise changes in the ECMO flow; the flow was reduced from baseline (100% ECMO flow) in steps of 10%, approximately every 10 min, until 70% of the baseline ECMO flow was reached, afterwards the flow was increased back to baseline following the same profile. In cases where the patients could not accommodate a 30% reduction in ECMO flow it was only reduced by a total of 20%. The interrelations between the set of peripheral and cerebral NIRS changes with the systemic variables were studied using two different approaches. The first approach used the complete measurement period and the ECMO flow when available as a parameter in the analysis. In the second approach, the signals were segmented in epochs of constant ECMO flow and the methods were applied separately to each epoch. In addition, in order to normalize the results to be comparable between patients, we estimated the ratio between the percentage of variance in the peripheral NIRS explained by the systemic variables and the percentage of variance of the cerebral NIRS explained by the systemic variables. We call this index the peripheral to cerebral haemodynamic ratio (PCHR). PCHR can be used to quantify the differences in the interrelations between both cerebral and peripheral circulation mechanisms versus systemic variations. PCHR values lower than 1 indicate that variations in the systemic variables are more likely to be reflected in the muscle than in the brain.

## Results

Table 4.1 shows PCHR for different ECMO flows and for the full measurement period. Results for the full measurement period show that only patient 4 presented a PCHR  >  1, indicating that the variations in the systemic variables were more likely to be reflected in the brain NIRS measurements rather than the peripheral in that patient; in the other patients the peripheral NIRS changes are more likely to be affected by variations in the systemic variables. In addition, patient 3, in contrast with the other patients, was the only one who, at every ECMO flow rate, consistently presented PCHR  <  1 and as it happened was the only patient in our group that the clinicians were confident to reduce the flow down to 70% from baseline. Table 4.2 shows which systemic variables contributed more to the changes in cerebral/peripheral NIRS variables, when analyzing the complete measurement period. HR and skin temperature were the systemic variables that most affected the cerebral and/or peripheral haemodynamic variables in the patients. MABP, CVP, skin temperature, SaO_2_ and ECMO affected the NIRS variables to a lesser extent.

**Table 4.1:** Ratio between the percentage of the variance in the cerebral haemodynamics and the peripheral haemodynamics explained by variations in the systemic variables (PCHR), for different ECMO flow percentages and the full measurement period

	Age (days)	ECMO flow percentage							Full period
		100%	90%	80%	70%	80%	90%	100%	
Patient 1	180	0.56	0.76	1.19	–	–	1.59	1.00	0.85
Patient 2	1	0.68	1.55	1.61	–	–	0.55	–	0.82
Patient 3	2	0.91	0.95	0.49	0.89	0.91	0.47	0.38	0.91
Patient 4	4	0.66	1.68	0.73	–	–	0.71	1.00	1.21
Patient 5	1,825	0.75	1.42	–	–	–	–	1.33	0.82

A PCHR lower than 1 indicates that the variations in systemic variables are more likely to be reflected in the muscle than in the brain. Spaces marked with a dash (–) indicate that it was not possible to perform the analysis due to the lack of measurements

**Table 4.2:** Systemic variables that presented a correlation higher than 0.5, in absolute value, with cerebral/peripheral haemodynamic variables represented by a C&P sign, respectively

Patient	HR	MABP	CVP	CoreT	SkinT	SaO_2_	RR	EtCO_2_	Flow
1	C	C&P	C		P				–
2	C&P	C	C	C	C&P	P	C&P	–	C&P
3	P	P		C&P	C&P	C&P	–	C	C&P
4	C&P		P	–	C&P	–			C&P
5	C&P	C	C&P		C&P	C&P			–

The blank spaces indicate no correlation, and a dash (–) indicates that the parameter was not used in the analysis due to lack of measurements or distortion by artefacts

## Discussion

PCHR  <  1, but close to 1, were observed in four out of five patients when analyzing the full measurement period. Earlier studies on neonates supported on ECMO indicate that autoregulation may be disrupted [1]; this can be the cause of PCHR values close to 1 as the brain haemodynamic and oxygenation changes will respond passively to systemic variations. In addition, when reducing ECMO flows it is expected that the peripheral circulation will be more affected by systemic changes than the brain circulation. At baseline level (100% ECMO flow) all patients reported a PCHR  <  1; when reducing the flow to 90% three out of five patients reported PCHR  >  1, indicating that systemic changes were more reflected in the cerebral circulation than in the peripheral circulation, while patient 5 could not accommodate extra reductions in the flow and was returned to baseline level. At 80% flow two out of four patients presented PCHR  >  1; furthermore, in this stage, three of the four patients could not accommodate more reductions in the flow and were returned to baseline level. Only patient 3 was able to accommodate a reduction to 70% in the ECMO flow and was the only patient who consistently presented PCHR  <  1. When returning ECMO flow to baseline level, three out of four patients presented PCHR  ≥  1. These results suggest that at low ECMO flows cerebral circulation is more vulnerable to changes in systemic variables and this effect is apparent in the most vulnerable patients.

In the population studied HR and skin temperature were the variables that most affected the cerebral and peripheral NIRS signals. Out of five patients, MABP was correlated with cerebral and peripheral haemodynamic variables in three and two patients, respectively. Out of four patients, SaO_2_ was correlated with cerebral and peripheral haemodynamic variables in three and two patients, respectively. Out of four patients EtCO_2_ was correlated with the cerebral haemodynamic variables in one patient. Furthermore, ECMO flow presented a strong correlation with cerebral and peripheral NIRS variables in all three patients with flow measurements. The lack of homogeneity in relation to the systemic variables that affect the cerebral or peripheral circulation in our study suggests differences in the clinical condition of each patient; however, due to the small patient numbers it is difficult to present a clinical hypothesis. Interestingly, and in contrast with these results, Tisdall et al. [9] in healthy adults found that changes in SaO_2_ and EtCO_2_ make a large contribution to changes in the cerebral NIRS TOI signal. In addition, CCA indicated that variation in the skin temperature strongly affects the cerebral and peripheral NIRS changes; conversely, Harper et al. [10] reported that a change in core temperature in adults can produce changes in blood flow due to changes in blood viscosity and metabolic rate (among other reasons). Whilst, Davis et al. [11] reported that changes in temperature produce changes in skin blood flow that can have a significant impact in the NIRS derived signals; therefore, special care should be taken when analyzing these results.

Several factors should be taken into account before interpreting the results provided by CCA. Among the limitations, the length of the signal under analysis and the presence of noise, non-linearities and nonstationarity can be cited. In order to overcome some of these problems, and compare the results between patients, normalization such as the PCHR ratio should be used; otherwise, the results should be interpreted carefully. Cerebral and peripheral haemodynamic and oxygenation changes are affected by multiple factors; hence underlying the necessity of measuring several systemic variables in order to obtain a general idea of the mechanisms affecting them. CCA is a useful tool to investigate this problem as it helps to assess and quantify the interrelation between a multiple set of variables, simultaneously.
